# HEALTH ACROSS BORDERS: A CROSS-NATIONAL COMPARISON OF IMMIGRANT HEALTH IN EUROPE

**DOI:** 10.1093/geroni/igac059.2092

**Published:** 2022-12-20

**Authors:** Mara Sheftel, Rachel Margolis, Ashton Verdery

**Affiliations:** Penn State University, Brooklyn, New York, United States; Western University, London, Ontario, Canada; The Pennsylvania State University, State College, Pennsylvania, United States

## Abstract

Although older immigrants are a growing share of the total population in many countries, evidence regarding health differentials by nativity in older adulthood remains underdeveloped. We examine whether foreign-born adults 50 and older in Europe are disadvantaged in terms of multiple health domains, what drives the potential immigrant health disadvantage, and whether such differences are contextually dependent or a general feature of the immigrant experience in Europe. We use the Survey of Health, Aging and Retirement in Europe (SHARE) to estimate physical, mental, and social health of middle age and older adults by nativity in 19 countries. We examine whether nativity-based health disparities can be attributed to demographic composition, socioeconomic factors, family and social support, and life course timing of migration. Last, we examine regional differences in nativity-based health disparities. We find that immigrants aged 50 and above in Europe are more likely to report fair/poor physical health, score worse on EURO-D depression scale, and are more likely to be lonely than the native-born. Socioeconomic status and age at migration partially explain these health differences, although immigrant health disparities remain after accounting for these and other factors. We document some contextual variation within Europe. Immigrants in Eastern, Western and Northern Europe are disadvantaged compared to native-born adults in those regions, while immigrants in Southern Europe are in comparable health to their native-born peers. This article offers new insights into the ways that aging immigrant populations will reshape older adult health profiles in a diverse array of countries.

